# Optimization of the Synthesis of Glycerol Derived Monoethers from Glycidol by Means of Heterogeneous Acid Catalysis

**DOI:** 10.3390/molecules23112887

**Published:** 2018-11-06

**Authors:** Elisabet Pires, José Ignacio García, Alejandro Leal-Duaso, José Antonio Mayoral, José Ignacio García-Peiro, David Velázquez

**Affiliations:** 1 Instituto de Síntesis Química y Catálisis Homogénea (ISQCH), CSIC-University of Zaragoza-Calle Pedro Cerbuna, 12, E-50009 Zaragoza, Spain; jig@unizar.es (J.I.G.); alduaso@unizar.es (A.L.-D.); mayoral@unizar.es (J.A.M.); 2 Departmento de Química Orgánica, Facultad de Ciencias, University of Zaragoza, Calle Pedro Cerbuna, 12, E-50009 Zaragoza, Spain; joseignacio.garcia.peiro@gmail.com (J.I.G.-P.); davidvelazquezojeda@gmail.com (D.V.)

**Keywords:** green solvent, glycerol, glycidol, glycerol ether, heterogeneous catalysis, acid catalyst, Bronsted acid, Lewis acid

## Abstract

We present an efficient and green methodology for the synthesis of glycerol monoethers, starting from glycidol and different alcohols, by means of heterogeneous acid catalysis. A scope of Brønsted and Lewis acid catalysts were applied to the benchmark reaction of glycidol and methanol. The selected catalysts were cationic exchangers, such as Nafion NR50, Dowex 50WX2, Amberlyst 15 and K10-Montmorillonite, both in their protonic form and exchanged with Al(III), Zn(II) and Fe(III). Thus, total conversions were reached in short times by using 1 and 5% mol catalyst loading and room temperature, without the need for excess glycidol or the presence of a solvent. Finally, these conditions and the best catalysts were successfully applied to the reaction of glycidol with several alcohols such as butanol or isopropanol.

## 1. Introduction

The vast majority of chemical industry processes rely on the use of conventional organic solvents. Although there are undisputable advantages in the use of these kinds of solvents, there are also important drawbacks, such as their (eco)toxicity, volatility and flammability. For this reason, developing cost-effective, harmless and environmentally benign alternative solvent systems is a research area of great interest.

Among the different proposals, glycerol itself and its derivatives are attracting a lot of attention as green and renewable solvents [1,2,3,4]. In particular, glycerol-derived monoethers (GMEs) have arisen as a promising option due to their chemical stability, tunable physical-chemical properties by just adjusting the nature of the substituent [5], relatively low ecotoxicology [6,7,8] and also due to the possibility of forming low melting mixtures (LMM) or Deep Eutectic Solvents (DES) [9].

When doing a search for potential applications for glycerol ethers in Scifinder Database, more than 7000 records dealing with patents involving the use of these compounds can be found. Thus, focusing on GMEs, the most common uses for these compounds are as additives in cosmetics or pharmaceuticals [10], and also in the formulation of dry-cleaning [11], cement formulation [12], cutting fluids [13], corrosion inhibitors [14], detergents [15], inks [16], herbicides and lubricants [17]. Particularly, 3-[(2-ethylhexyl)oxy]-1,2-propanediol, known as Sensiva^®^SC50, is one of the GMEs used in skin creams.

For all these reasons, it is of great importance to develop sustainable methodologies for obtaining glycerol monoethers (GMEs). Lemaire and co-workers have beautifully reviewed the synthesis of glycerol ethers, starting from both glycerol and platform molecules, such as glycidol, epichlorohydrin and 3-chloropropane-1,2-diol, which can in turn be prepared from glycerol [18]. As mentioned in this work, scarce data on the production of GMEs is found despite the numerous potential applications of glycerol alkyl ethers [19,20,21].

Addressing the synthesis of GMEs, the direct synthesis from glycerol has been envisaged but the difficulty in selectively reacting just one of the hydroxyl groups of glycerol is the main drawback of this approach. Thus, the best results described until now in the reaction of glycerol with several alcohols were presented by Jerôme et al. [22]. They described 70% selectivity towards GMEs using homogeneous Lewis catalysts, such as Bi(OTf)_3_. Recently Henriques et al. [23] described a similar selectivity in the reaction of glycerol and ethanol using a heterogeneous catalyst, namely USY, HZSM-5 and H-Beta zeolites. In both cases, obtaining high selectivity of GMEs is achieved by the use of high reaction temperatures and an excess of glycerol, which can be difficult to separate after the reaction is completed.

Thus, the use of building blocks usually improves the selectivity of the process and allows to obtain mono-, di- and triethers with different alkyl chains in a controlled way without the need for excess of starting reagent.

Some works have been published where GMEs have been obtained from glycidol with excellent conversions and selectivities by using homogeneous Lewis acids as catalysts [24,25]. The authors described high glycidol conversions and product selectivities over 90% with the use of Bi(OTf)_3_ and Al(OTf)_3_ as catalysts. A very low catalyst loading of 0.01% is used and the reaction is carried out at 80 °C. The use of these catalysts avoided the oligomerization of glycidol to a major extent, which could be an advantage compared to the use of basic catalysis [26], but the elimination of the catalyst from the reaction medium can be considered as a drawback as it implies the loss of parts of the GMEs that possess a non-negligible water solubility. This methodology has been improved by the same authors by using heterogeneous acid catalysts, namely Nafion NR50 [27]. Total glycidol conversions are described with a low catalytic loading (0.5%) and product yields in between 86% for isopropanol and 6% for 2-ethylhexan-1-ol.

We recently presented a work dealing with the synthesis of GMEs starting from glycidol and several alcohols in the presence of both homogeneous and heterogeneous basic catalysts [26]. Although very good yields were achieved, we can consider some drawbacks that should be overcome in order to improve the sustainability of the process. First, the best results were described with the use of 20% mol of alkaline hydroxides that have to be neutralized in reaction media with subsequent salt formation. Also in the case of using more hindered alcohols or less reactive ones, the oligomerization of glycidol took place in a non-negligible extent. That is why we envisaged the possibility of exploring the use of heterogeneous acid catalysts as an alternative to our previous results.

Here, we propose an efficient and green methodology for the synthesis of glycerol monoethers starting from glycidol and different alcohols (methanol, butanol and isopropanol) by means of heterogeneous acid catalysis. This methodology intends to overcome some drawbacks of previously described synthetic procedures such as the use of excess glycidol or the use of homogeneous catalyst, thus improving the sustainability of the synthesis.

## 2. Results and Discussion

### 2.1. Reaction of Glycidol and Methanol by Means of Heterogeneous Brønsted Acid Catalysis

**Scheme 1:**
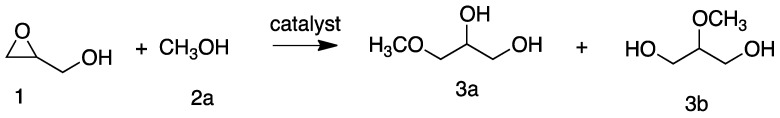
Reaction of glycidol (**1**) with methanol (**2a**) used as the benchmark reaction.

First, the reaction conditions were optimized in the benchmark reaction of glycidol (**1**) with methanol (**2a**) by using Dowex 50WX2 as catalyst (Scheme 1). Dowex 50WX2 is a sulfonic resin that has been commonly and successfully used in catalysis [28,29,30]. The influence on both glycidol conversion and product selectivity (**3a** and **3b**) was studied by modifying the loading of catalyst (5, 10 and 20% mol in respect to glycidol) and the reaction temperature (25 and 65 °C). In all cases methanol was used as the solvent (methanol: glycidol molar ratio 15:1). The reaction was monitored by means of Gas Chromatography (GC) using diglyme as the internal standard.

**Figure 1:**
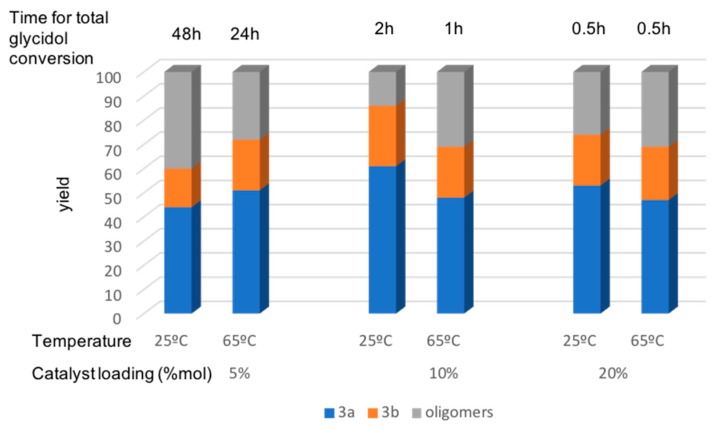
Influence of catalyst loading and reaction temperature in glycidol (**1**) conversion and product yields in the reaction of glycidol and methanol catalyzed with Dowex 50WX2.

When using 10% or 20% mol catalyst loading, total conversions of glycidol were reached in less than 2 h regardless of the reaction temperature (Figure 1). Thus, using 20% of Dowex 50WX2, glycidol was totally converted in only 0.5 h. When lowering the catalyst loading to 5%, the reaction was dramatically slowed down, and 24−48 h was necessary to complete the reaction.

Apart from glycidol conversion, yields of both products **3a** and **3b** were determined (Figure 1). As can be seen, the ratio **3a/3b** is almost constant (70/30) and not dependent on the reaction conditions. But the selectivity of the reaction, and thus the formation of by-products (mostly glycidol oligomers), is greatly influenced by the reaction temperature and catalyst loading. Thus the optimal conditions in order to obtain a higher selectivity is to use a 10% catalyst loading and 25 °C reaction temperature. These conditions were applied to the rest of the catalysts.

Once the best reaction conditions were established (10% mol catalyst and 25 °C), a selection of solid Brønsted acid catalysts was made in order to study the influence of the nature of the acid site in the reaction results. For this purpose, synthetic resins with aryl, alkyl and perfluoroalkyl sulfonic groups (Amberlyst A15, Dowex 50WX2, Deloxan I/9, Nafion NR50 and Aquivion PW79S) and carboxylic sites (Dowex CCR-2) were selected, together with a natural clay K10-H^+^ Montmorillonite.

**Figure 2:**
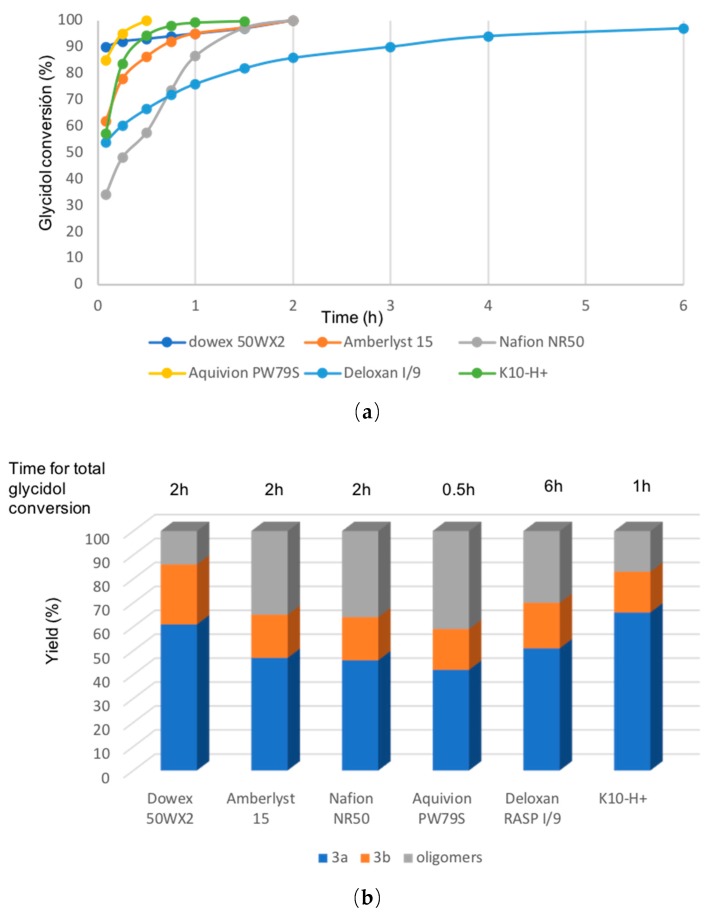
Influence of the nature of the catalyst on glycidol (**1**) conversion (**a**) and products selectivity (**b**).

Catalyst with carboxylic groups Dowex CCR-2 provided very poor glycidol conversions (14%) in long reactions times (24 h), this catalyst being discarded. Comparing the rest of the catalysts, an increase in oligomers formation was observed when using more acidic solids such as Nafion NR50 or Aquivion PW79S compared to the results obtained with Dowex 50WX2 (Figure 2b). The use of Amberlyst 15, a more crosslinked resin, also worsened the selectivity of the reaction towards **3a** and **3b**, and the activity of the alkyl sulfonic resin Deloxan I/9 was lower as expected (Figure 2a), thus 6 h were necessary to achieve total glycidol conversion. With all of these catalysts, a ratio **3a**/**3b** of 70:30 was achieved and the product selectivity was maintained along the reaction.

Finally, acidic K10 Montmorillonite provided very good results, as 80% yield of the products was achieved in only 1 h reaction time, and with a higher regioselectivity (ratio **3a**/**3b** 80:20). This catalyst was the best of the tested solids in activity and product selectivity. Above this, its natural origin improves the sustainability of the process.

### 2.2. Reaction of Glycidol and Methanol by Means of Heterogeneous Lewis Catalysis

Based on the studies presented by Cuccinello et al. [24,25], we decided to test some heterogeneous Lewis acid catalysts in order to explore the possibility of improving the product selectivity with respect to the ones obtained with Brønsted solid K10-H^+^. As it has been mentioned, the fact of obtaining two regioisomers, **3a** and **3b**, should be addressed as an advantage if the mixture is used as a solvent due to the similar physical-chemical characteristics of both products, but it is a great drawback if only one of the products is intended for use, due to the difficulty of the separation of these compounds due to their chemical similarity. The good results obtained by using K10-H+ as a catalyst prompted us to try the same catalysts exchanged with Al^+3^ (K10-Al), Zn^+2^ (K10-Zn) or Fe^+3^ (K10-Fe), both dried at 120 °C and calcined at 550 °C. Previous IR studies of adsorbed pyridine, as a probe molecule, on exchanged Montmorillonites, showed the presence of both Brønsted and Lewis sites in dried solids at 120 °C and an increase of Lewis acidity upon calcination at 550 °C [31]. An approximate comparison of the relative acidity of several clays has been carried out by comparing the intensity of the IR peaks of pyridine coordinated to Br*ø*nsted and Lewis sites.

The possibility of using 10% mol of these catalysts, as in the case of K10-H^+^, was first considered, but the lower functionalization of K10-Al (0.18 mmol Al g^−1^), K10-Fe (0.2 mmol Fe g^−1^) and K10-Zn (0.28 mmol Zn g^−1^) in respect to K10-H^+^ (0.59 mmol H^+^ g^−1^) implies the use of an amount of solid that makes the work up of the reaction difficult. That is why we decided to start the catalytic test with 5% mol catalyst.

Table 1 gathers the results of the reaction of glycidol (**1**) with methanol (**2a**) at room temperature, and as it can be seen, the lower activity and higher regioselectivity of calcined solids (entry 4 to 6) agree with the lower number of acid sites and the increase of the Lewis/Br*ø*nsted sites ratio.

Thus, a different behavior is observed for each catalyst but with a common trend, upon calcination the reaction was slowed down in all the cases. Thus, the presence of Br*ø*nsted acid sites accelerates the reaction. It is also noteworthy that K10-Zn (entry 3) and calcined clays (entries 4–6) greatly improved the regioselectivity (**3a**/**3b**) with respect to K10-H^+^. In this case, Lewis acid sites favor the attack of methanol to the less substituted carbon of the epoxide ring. This is also observed when comparing the **3a**/**3b** ratio in the reactions where the catalysts are activated at 120 °C (Table 1 entries 1 to 3). As Lewis/Br*ø*nsted increases (K10-Zn > K10-Al > K10-Fe) [31], the **3a**/**3b** ratio also follows the same trend.

**Figure 3:**
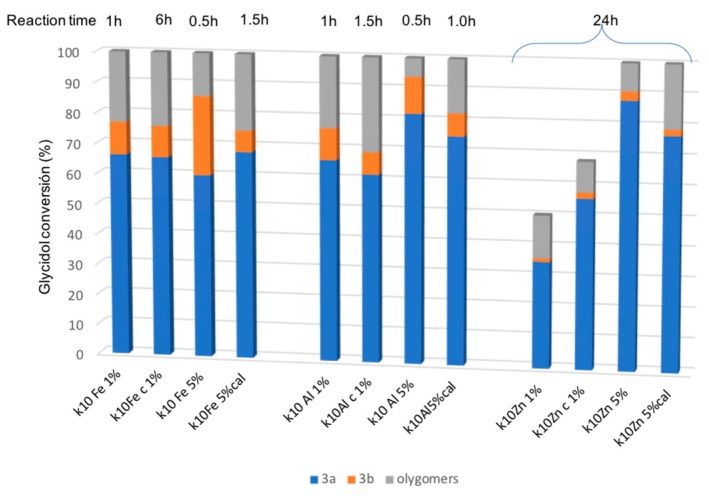
Reaction of glycidol (**1**) with methanol (**2a**) catalyzed by 1% mol and 5% mol K10.

The good results obtained with these solids prompted us to try to reduce the amount of catalyst. Thus the reactions were tested using 1% mol of solid. A comparison of the results with 1% and 5% catalyst are shown in Figure 3.

As it can be seen in Figure 3, a reduction in the amount of catalyst increases the time for total glycidol conversions in all the cases. It is noteworthy that in the case of K10-Zn, low glycidol conversions are achieved even at 24 h reaction time when using 1% catalyst. In the case of K10-Fe and K10-Al, a higher amount of byproducts are formed when reducing the amount of catalyst. Finally, product yields are also affected. Thus for K10-Al, the yield of **3a** + **3b** is reduced from 94% when using a 5% mol catalyst to 80% when using 1% mol. For K10-Zn the effect is even more dramatic with a drastic decrease in yields from 91% yield to 36%.

**Figure 4:**
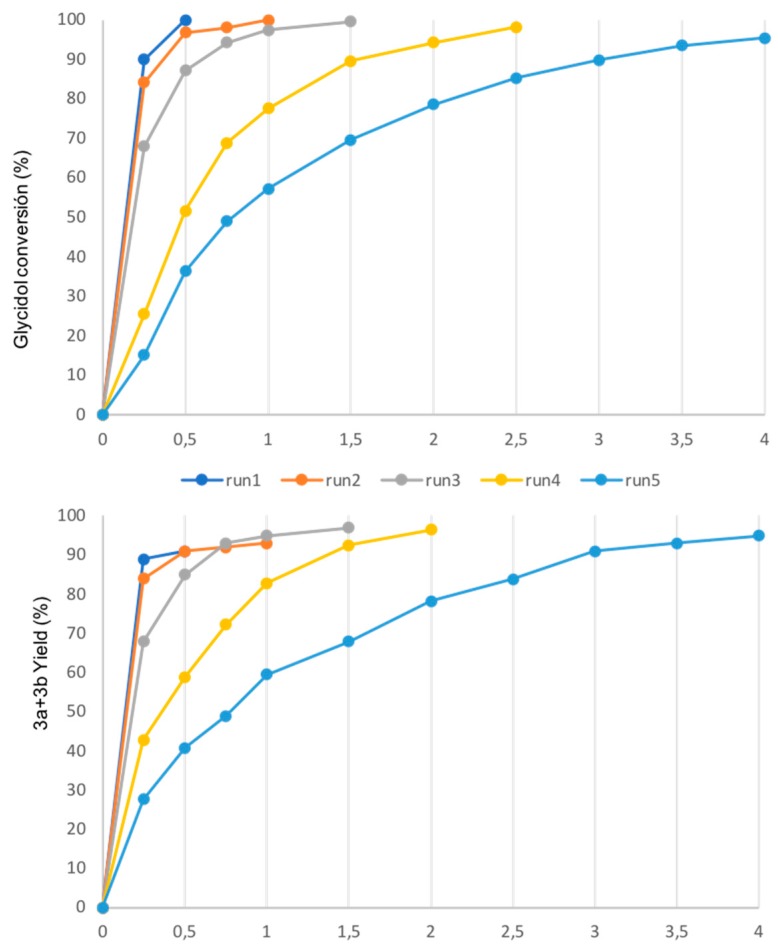
Recoverability of K10-Al (5% mol) in the reaction of glycidol (**1**) with methanol (**2a**).

The analysis of the data in Figure 3 shows that the best results for the reaction of glycidol with methanol are achieved when using 5% mol K10-Al treated at 120 °C, both in terms of glycidol conversion and product selectivity. Thus, these reaction conditions were applied in the study of the recoverability of the catalyst (Figure 4).

When recovering K10-Al two consecutive runs can be done with no loss of activity. But from run 3, a progressive loss of activity is observed. However, total conversions of glycidol are achieved at room temperature in moderate reaction time. Finally, it is noteworthy that in all the cases a product selectivity over 90% is maintained.

### 2.3. Reaction of Glycidol with Several Alcohols for the Synthesis of GMEs

**Scheme 2:**
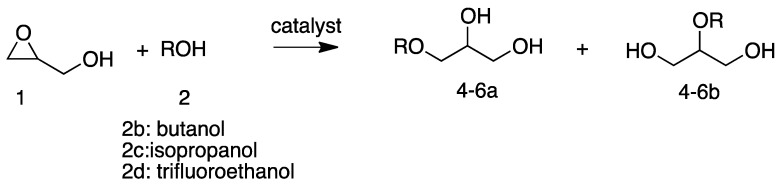
Reaction of glycidol (**1**) with several alcohols (**2**).

In order to demonstrate the applicability of these catalytic systems to the synthesis of GMEs, the reaction of glycidol with other alcohols was performed (Scheme 2). Again, K10 Montmorillonites were chosen due to the good results provided in the reaction with methanol.

Unfortunately, reactions using trifluoroethanol (**2d**) provided very poor yields in a long reaction time (e.g., with K10-H^+^, 5% yield was achieved in 6 h reaction time). These results are attributed to the low nucleophilicity of this alcohol. However, as it can be seen in Table 2, the catalysts provided good results when using isopropanol or butanol. These results being the best described up to now for the synthesis of GMEs with heterogeneous catalysis.

### 2.4. Comparison of Synthetic Methodologies for Obtaining GMEs

In order to be able to select the best methodology for the synthesis of the glycerol monoethers starting from glycidol for each alcohol (**2a**–**2c**), we have gathered in Table 3 the best results reported in this manuscript together with the best results published up to now for these reactions.

This comparative table can be a useful tool for designing or scaling up the synthesis of GMEs. In addition, several parameters can be tuned in order to obtain either better conversions or selectivities, or to prioritize lower reaction temperatures or catalyst loading depending on its price and recoverability.

As it can be seen, results provided by K10 Montmorillonites can improve the sustainability of the synthetic process for the obtaining of GMEs as good yields and selectivities can be achieved at room temperature. Although a higher loading of the catalyst is needed, the possibility of its reuse is also an advantage compared to the use of homogeneous catalysts such as KOH or Al (OTf)_3_.

## 3. Materials and Methods

Gas chromatography was carried out in a Hewlett Packard 5890 and 7890 series II Gas Chromatograph using a column Zebron ZB-Wax 30 m × 0.25 mm × 0.25 μm for the determination of the results of the reaction of glycidol with methanol, and for the rest of the alcohols, a column of phenyl silicone 5.5% (Zebron ZB-5HT Inferno 30 m × 0.25 mm × 0.25 μm) was used. Helium was used as a carrier gas, and the detection was done with a flame ionization detector (FID).

Glycidol, diglyme, Dowex 50WX2, K10 Montmorillonite, Aquivion PW79S and Amberlyst 15 were purchased from Sigma-Aldrich (St. Louis, MO, USA). Methanol and isopropanol, were purchased from Scharlab (Barcelona, Spain). Butanol, 2,2,2-trifluoroethanol and Nafion NR50 were purchased from Alfa Aesar (Haverhill, MA, USA).

### 3.1. Preparation of the Catalysts

Prior to the reaction, commercial sulfonic resins were dried under vacuum at 50 °C for two days and K10 clays were dried at 120 °C overnight or calcined at 550 °C.

Preparation and activation of K10-Al, K10-Fe and K10-Zn is described elsewhere [31].

### 3.2. Reaction Procedure for the Synthesis of Glycerol Monoethers

The appropriate amount of alcohol (15:1 with respect to glycidol), the catalyst (5, 10 or 20% mol with respect to glycidol) and diglyme as internal standard (15% *w*/*w* with respect to glycidol) were placed into a round bottomed flask. The reaction was stirred and heated at the desired temperature (65 or 25 °C) under argon. Then, glycidol (4.35 mmol) was added dropwise for 15 min. The reaction was monitored at different times by extracting samples that were filtered and diluted with methanol prior to injection in GC. After total consumption of glycidol, the heterogeneous catalysts were just filtered off. Conversions and yields were determined by GC.

All the alcohols were dried and distilled over calcium hydride prior to use.

## 4. Conclusions

A simple methodology for the synthesis of glycerol monoethers from glycidol and several alcohols has been presented. The use of heterogeneous catalysts, such as exchanged Montmorillonites, room temperature, the lack of need for any excess glycidol or additional solvents, and obtaining good yields and selectivities makes this process comply with the principles of green chemistry. The influence of catalyst loading and the reaction temperature was studied, as well as the influence of the nature of the catalyst and the alcohol. Brønsted catalysts provided good glycidol conversions in short times, but selectivity towards GMEs strongly depended on the catalyst. Thus, Dowex 50WX2 and K10-H^+^ provided the best yields. Exchanged K10 Montmorillonites showed a good activity in the reaction of glycidol and several alcohols, these results being comparable to previous results in the literature. Elimination of Bronsted acidity by calcination in exchanged K10 clays slowed down the reactions but a slight increase in regioselectivity was obtained.

## Figures and Tables

**Table 1:** Results of the reaction of glycidol (**1**) with methanol (**2a**) at room temperature catalyzed by exchanged K10-Montmorillonites ^1^.

Entry	Catalyst	T (°C) Activation	Time (h) ^2^	Products Yield	3a/3b
1	K10-Al	120	0.5	94	82:18
2	K10-Fe	120	0.5	86	70:30
3	K10-Zn	120	18	91	93:7
4	K10-Al-calc	550	1.5	84.9	89:11
5	K10-Fe-calc	550	1.5	74.8	91:9
6	K10-Zn-calc	550	24	79.1	90:10

^1^ A ratio **2a**/**1** of 15 mol and 5% mol catalyst are used, **1** added dropwise; ^2^ time for total conversion of glycidol is considered.

**Table 2:** Results of the reaction of glycidol with other alcohols at room temperature catalyzed by exchanged K10-Montmorillonites **^1^**.

Alcohol	Catalyst	Time (h) ^2^	Products Yield	4–5a/4–5b
**2b**	K10-H^+^	0.5	70	83:17
**2b**	K10-Al	0.5	79	76:24
**2b**	K10-Zn	24	65 ^3^	88:12
**2c**	K10-H^+^	2	78	91:9
**2c**	K10-Al	1	75	85:15
**2c**	K10-Zn	24	72	95:5

**^1^** A ratio **2**/**1** of 15 mol are used and 5% mol catalyst is used in the case of K10-Al and K10-Zn and 10% in the case of K10-H^+^, **1** added dropwise; ^2^ time for total conversion of glycidol is considered; ^3^ 85% glycidol conversion after 24 h.

**Table 3:** Comparison of the synthetic methodologies for the synthesis of GMEs (**3**–**5a** and **3**–**5b**).

Alcohol	Catalyst	Catalyst Loading (% mol)	Alcohol/Glycidol Molar Ratio	Time (h)	Reaction T (°C)	Conversion (Selectivity)	a/b	Ref.
**2a**	KOH ^1^	20	15	2	65	100 (93)	100:0	[26]
**2a**	Al(OTf)_3_	0.01	9	1	80	100 (94)	66:34	[24]
**2a**	Nafion NR50	0.5	9	1	80	85 (79)	77:23	[27]
**2a**	Nafion NR50	10	9	2	25	100 (67)	72:28	
**2a**	K10-H^+1^	10	15	0.5	25	100 (83)	80:20	
**2a**	K10-Al ^1^	5	15	0.5	25	100 (94)	82:18	
**2a**	K10-Zn ^1^	5	15	24	25	100 (91)	93:7	
**2b**	KOH ^1^	20	15	1.5	65	100 (60)	100:0	
**2b**	Al(OTf)_3_	0.01	9	1	80	94 (91)	66:34	[24]
**2b**	Nafion NR50	0.5	9	15	80	39 (37)	72:28	[27]
**2b**	K10-H^+1^	10	15	0.5	25	100 (70)	83:13	
**2b**	K10-Al ^1^	5	15	0.5	25	100 (79)	76:24	
**2c**	K10-Zn ^1^	5	15	24	25	85 (65)	88:12	
**2c**	KOH ^1^	20	15	2	65	100 (30)	100:0	
**2c**	Al(OTf)_3_	0.01	9	1	80	85 (87)	66:34	[24]
**2c**	Nafion NR50	0.5	9	15	80	95 (86)	78:22	[27]
**2c**	K10-H^+1^	10	15	2	25	100 (78)	91:9	
**2c**	K10-Al ^1^	5	15	1	25	100 (75)	85:15	
**2c**	K10-Zn ^1^	5	15	24	25	100 (72)	95:5	

^1^ Glycidol (**1**) is added dropwise.
